# Inhibitory effects of *Stemona tuberosa* on lung inflammation in a subacute cigarette smoke-induced mouse model

**DOI:** 10.1186/1472-6882-14-513

**Published:** 2014-12-20

**Authors:** Hyeonhoon Lee, Kyung-Hwa Jung, Soojin Park, Yun-Seo Kil, Eun Young Chung, Young Pyo Jang, Eun-Kyoung Seo, Hyunsu Bae

**Affiliations:** Department of Korean Medicine, College of Korean Medicine, Kyung Hee University, #1 Hoeki-dongDongdaemoon-gu, Seoul, 130-701 Republic of Korea; Department of Physiology, College of Korean Medicine, Kyung Hee University, #1 Hoeki-dongDongdaemoon-gu, Seoul, 130-701 Republic of Korea; Global Top5 Research Program, College of Pharmacy, Ewha Womans University, Seoul, 120-750 Korea; Department of Life and Nanopharmaceutical Science, College of Pharmacy, Kyung Hee University, #1 Hoeki-dongDongdaemoon-gu, Seoul, 130-701 Republic of Korea

**Keywords:** CS, Stemona tuberosa, BALF, COPD, TNF-α, IL-6

## Abstract

**Background:**

*Stemona tuberosa* has long been used in Korean and Chinese medicine to ameliorate various lung diseases such as pneumonia and bronchitis. However, it has not yet been proven that *Stemona tuberosa* has positive effects on lung inflammation.

**Methods:**

*Stemona tuberosa* extract (ST) was orally administered to C57BL/6 mice 2 hr before exposure to CS for 2 weeks. Twenty-four hours after the last CS exposure, mice were sacrificed to investigate the changes in the expression of cytokines such as tumor necrosis factor-alpha (TNF-α) and interleukin-6 (IL-6), chemokines such as keratinocyte-derived chemokine (KC) and inflammatory cells such as macrophages, neutrophils, and lymphocytes from bronchoalveolar lavage fluid (BALF). Furthermore, we compared the effect of ST on lung tissue morphology between the fresh air, CS exposure, and ST treatment groups.

**Results:**

ST significantly decreased the numbers of total cells, macrophages, neutrophils, and lymphocytes in the BALF of mice that were exposed to CS. Additionally, ST reduced the levels of cytokines (TNF-α, IL-6) and the tested chemokine (KC) in BALF, as measured by enzyme-linked immunosorbent assay (ELISA). We also estimated the mean alveolar airspace (MAA) via morphometric analysis of lung tissues stained with hematoxylin and eosin (H&E). We found that ST inhibited the alveolar airspace enlargement induced by CS exposure. Furthermore, we observed that the lung tissues of mice treated with ST showed ameliorated epithelial hyperplasia of the bronchioles compared with those of mice exposed only to CS.

**Conclusions:**

These results indicate that *Stemona tuberosa* has significant effects on lung inflammation in a subacute CS-induced mouse model. According to these outcomes, *Stemona tuberosa* may represent a novel therapeutic herb for the treatment of lung diseases including COPD.

## Background

Cigarette smoke (CS) comprises more than 4,500 compounds, including human carcinogens and many toxins such as nicotine, carbon monoxide [1]. CS, which is one of the risk factors of chronic obstructive pulmonary disease (COPD), increases various respiratory symptoms resulting from functional lung abnormalities such as a reduced forced expiratory volume in 1 sec (FEV1) [2]. CS induces lung inflammation through different types of mechanisms, including reduced fibroblast proliferation and migration, and genetic mutations in the lung [3–6]. Furthermore, various types of inflammatory cells, cytokines and chemokines are widely studied with regard to the activation of acute or chronic inflammation in the lungs, which may be important factors for understanding the mechanisms of lung inflammation and developing new approaches to treat lung diseases including COPD [7].

When exposed to CS, cytokines such as tumor necrosis factor-alpha (TNF-α) and interleukin-6 (IL-6) and the chemokine, keratinocyte-derived chemokine (KC) which is a neutrophil chemoattractant, were found to be elevated in the bronchoalveolar lavage fluid (BALF) of mice [8]. Inflammatory cells including macrophages, neutrophils, and lymphocytes also accumulated in BALF. These inflammatory cells release inflammatory substances such as enzymes that destroy collagen and elastin and stimulate mucosal secretions in the lung tissues, resulting in lung diseases like emphysema, chronic bronchitis, and COPD [1].

Meanwhile, COPD is a major public health problem worldwide. By definition, COPD is characterized by continued airflow limitation that is progressive and is associated with an increasing chronic inflammatory response in the airways and the lung to hazardous particles or gases, including CS. COPD has a higher risk of mortality than other diseases such as angina, respiratory infection, fractures, myocardial infarction, and osteoporosis [9]. In 2020, COPD is expected to rank fifth in terms of the burden of disease and will likely rank third as a cause of mortality [10].

Roflumilast, a common therapy for lung diseases such as asthma and COPD, has been shown to inhibit the increased secretion of cytokines such as TNF- α and interferon-gamma (INF-γ), and chemokines such as CCL2, CCL4, CXCL1, CXCL8, and CXCL10 in experimental studies of lung inflammation [11–13]. Roflumilast is generally used for treatment of COPD patients with reliable evidence of clinical trials [14, 15]. Based on these results, roflumilast was used in our study as a positive control.

*Stemona tuberosa* has long been used as a therapeutic herb in Korean and Chinese medicine for the treatment of lung diseases. There have been some reports on the antifungal or antibacterial effects of *Stemona tuberosa* [16–18], however, there have been only a few studies on its inhibitory effects on lung inflammation. It has also been reported that *Stemona tuberosa* has a significant effect on components of the respiratory system such as the larynx and the lungs [19, 20]. In this study, we investigated the effect of *Stemona tuberosa* on lung inflammation via a subacute CS-induced mouse model. We examined the therapeutic effects of ST in terms of both immunological changes such as decreased cytokines (TNF-α, IL-6) and chemokine (KC), and morphological changes in the lung microenvironment.

## Methods

### Animals

Groups of five six-week-old female C57BL/6 mice were purchased from Charles River Korea (Seoungnam, South Korea). All mice were kept under pathogen-free conditions with air conditioning and a 12 hr light/dark cycle. In addition, all mice had free access to food and water during the experiments. This experimental study was approved by the Institutional Animal Care and Use Board of Kyung Hee University (KHUASP (SE)-12-015).

### Preparation of Stemona tuberosa and phytochemical analysis

ST was purchased from Sun Ten (Sun Ten Pharmaceutical Co., Ltd. Taiwan). According to the manufacturer’s procedure, water extraction of *Stemona tuberosa* was performed. At first, the *Stemona tuberosa* was water extracted at 100°C for 60 min. And then, the remains of the herbs and impurities were separated from the extracted liquid as a filtration separation process. The water extracts were then spray dried and corn starch was added as an excipient to stabilize the concentrated herbal products (final ratio of water extracts *vs* starch is 7:3, Batch No: 110410). After these procedures, the final product of *Stemona tuberosa* extract (ST) was produced in Sun Ten. ST was carefully measured to prepare the necessary weights for each treatment group and then dissolved in distilled water (DW) for 30 min at room temperature.

**Figure 1:**
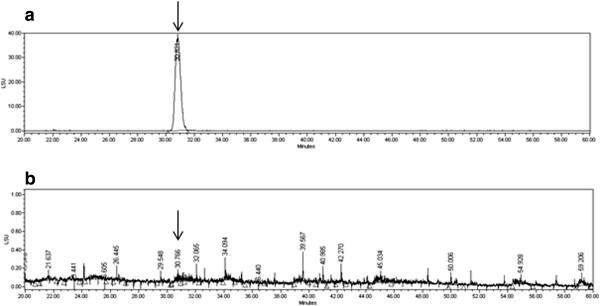
**The HPLC analysis of the standard material to ST.** Phytochemical analysis was performed by HPLC as described in the Methods section. Tuberostemonine N was utilized as an authentic standard (arrows) and found that the concentration of tuberostemonine N in ST was calculated to be 0.3 mg/g (yield 0.03%). **a)** HPLC-ELSD analytical data of tuberostemonine N, **b)** HPLC-ELSD analytical data of ST.

*Stemona tuberosa* extract (ST) is one of the herbal extracts in PM014, which are well-standardized herbal formula and has been investigated for treating lung diseases recently [21]. PM014 has been already approved for the Investigation New Drug (IND) application from Ministry of Food and Drug Safety, Republic of Korea (ID:20130030575) through quantitating standard materials by high-performance liquid chromatography (HPLC) analysis. Accordingly, ST can be considered as a standardized plant extract.For the phytochemical analysis of ST, HPLC was performed. The ST was accurately weighed to 10.53 mg and then dissolved in 2 ml of methanol. The sample was treated with ultrasonic waves for 30 min, and the supernatant of the sample was then filtered through a 0.45-μm syringe filter. For the quantitative analysis of ST, tuberostemonine N, one of the known alkaloid constituents of ST, was isolated from the ethyl acetate extract of ST by repeated column chromatography. HPLC analysis were conducted using a Waters system (Waters Co., Milford, MA, USA) with a 2424 ELS detector and a 1525 binary HPLC pump. A Waters Millennium 32 System (Waters Co., Milford, MA, USA) was used for data acquisition and integration. The samples were analyzed by reverse phase (C18) HPLC analysis (Agilent prep-C18 Scalar, 4.6 × 250 mm i.d., 5 μm, flow rate: 0.5 ml/min) using a gradient solvent system of acetonitrile containing 0.05% triethylamine (A) and water (B) as follows: 40% A at 0–10 min, 40 → 50% A at 10–20 min, 50% A at 20–40 min, 50 → 40% A at 40–50 min and 40% A at 50–60 min. The drift tube temperature for ELSD was set at 60°C, with 50 psi of pressure of the nebulizing nitrogen gas. The ELSD generates a signal in direct proportion to the quantity of the analyte present. Using this method, the concentration of tuberostemonine N in ST was calculated to be 0.3 mg/g (yield 0.03%) as described in (Figure 1).

### High Performance Liquid Chromatography – electrospray tandem mass spectrometry (HPLC-ESI-MS) analysis

The HPLC (High performance liquid chromatography) system operated by Chromeleon (Thermo Fisher Scientific, USA) and consisted of Dionex model P680 HPLC pump, a ASI-100 Automated Sample injector and a UVD 340U. The Brownlee Analytical C18 column (4.6 × 250 mm, 5 μm) was selected for the analysis. The monitoring wavelength was set to 254 nm. The mobile phase was comprised of acetonitrile with triethylamine (0.1%, solvent A) and water (solvent B). All solvents were filtered through a 0.45 m filter. The gradient program was 0–10 min, 40% of solvent A; 10– 12 min, linear from 40 to 50% of solvent A; 12–28 min, 50% of solvent A; 28– 30 min, linear from 50 to 40% of solvent A, at a flow rate of 0.6 mL per min. The injection volume was 10 μL.

AccuTOF® single-reflectron time-of-flight mass spectrometer was operated with Mass Center system version 1.3.7b (JEOL, USA) and was equipped with an ESI source (Electrospray ionization, JEOL, USA). In the positive ion mode, the atmospheric pressure interface potentials were typically set to the following values: orifice 1 = 80 V and ring lens and orifice 2 = 10, 5 V, respectively. The ion guide potential and detector voltage were set to 2000 V and 2300 V, respectively. ESI parameters were set as follows: needle electrode = 2000 V, nitrogen gas was used as a nebulizer, desolvating and their flow rate were1 and 3 L/min, desolvating chamber temperature = 250°C, orifice 1 temperature = 80°C. Mass scale calibration was accomplished with Yokudelna (Koyo Science Co., LTD., Japan) for accurate mass measurements and calculations of the elemental composition. MS acquisition was set with a scan range of *m/z* 50 to 1500.

### A mouse model of subacute CS-induced lung inflammation and drug treatment

**Figure 2:**
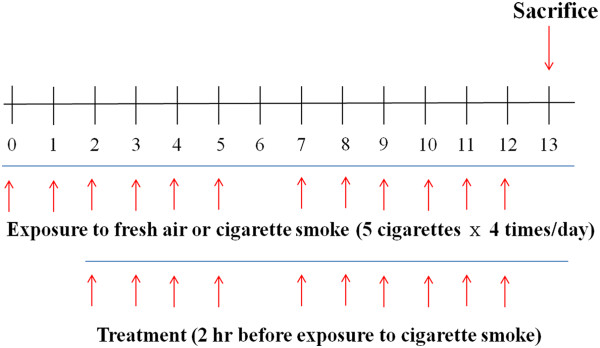
**Experimental design of subacute cigarette smoke-induced lung inflammation mouse model.** Female C57BL/6 mice were exposed (whole body) to the smoke of five cigarettes, four times per day with 30 min smoke-free intervals, on 6 days per week for 2 weeks. Mice were orally treated with DW on days 0 and 1, and DW, roflumilast (5 mg/kg), or ST (50, 100, and 200 mg/kg) on days 2 to 5, and 7 to 12. On day 13, mice were sacrificed to acquire BALF and lung tissues for other analyses.

Female C57BL/6 mice were exposed to either fresh air (as a control group) or CS generated by 3R4F Kentucky Reference Cigarettes (University of Kentucky, Lexington, KY). As the subacute CS-induced lung inflammation mouse model used here was based on previous studies [22, 23], mice were exposed over their entire bodies to the smoke of five cigarettes, four times per day with 30 min smoke-free intervals, on 6 days per week for 2 weeks. They were dosed with DW on days 0 and 1, and DW or ST on days 2 to 5 and 7 to 12 as described in (Figure 2). The mice were treated with 5 mg/kg dose of roflumilast (Santa Cruz Biotechnology, Inc., CA, USA) orally as a positive control on the same days as ST, and different doses of ST (50, 100, and 200 mg/kg) via oral injection 2 hr before exposure to CS. On day 13, the mice were sacrificed to obtain BALF and lung tissues for confirming this mouse model is successful and analyzing the results.

### Analysis of bronchoalveolar lavage fluid (BALF)

BALF was collected by the infusion and extraction of 1 ml of ice cold phosphate buffered saline (PBS). This procedure was repeated three times, and the lavages were pooled. The recovered BALF (70–80%) was centrifuged at 1,300 rpm for 10 min. The cell pellets were resuspended in 1 ml PBS and adhered to glass slides using cytocentrifugation. The total viable cell counts were determined in a hemocytometer using the trypan blue exclusion method. Differential counts of macrophages, neutrophils, and lymphocytes were determined on Diff-Quick-stained (Life Technologies, Auckland, New Zealand) cytospin smears of BALF samples (5 × 10^5^ /200 μl cells) from individual mice. BALF samples were then centrifuged and the supernatants were kept at -80°C. The results are expressed as the total cell number × 10^4^.

### Assessment of protein, cytokines and chemokines in BALF using Enzyme-Linked Immunosorbent Assay (ELISA)

Protein concentrations were determined using a BCA kit (Pierce Biotechnology Inc., IL, USA). A total 25 μl of the centrifuged BALF samples was placed into the microplate well, and then 200 μl of the working reagent was added to the each well. After incubation at 37°C for 30 min, the optical density was measured at 590 nm in a microplate reader (SOFT max PRO, version 3.1 software, Sunnyvale, CA, USA). The concentrations of cytokines (TNF-α, IL-6) and the chemokine (KC) were measured with a quantitative sandwich enzyme-linked immunoassay kit (BD, San Diego, CA, USA) following the manufacturer’s instructions. Microtiter plates (96-well, Costar, Corning, NY, USA) were incubated overnight at 4°C with anti-mouse TNF-α, IL-6, and KC monoclonal antibodies in coating buffer. The wells were then washed with PBS containing 0.05% Tween-20 (Sigma-Aldrich Co., St. Louis, MO, USA) and blocked with 5% FBS and 1% BSA in PBS. Subsequently, each well was loaded with 100 μl of BALF and incubated for 2 hr at room temperature. The wells were then incubated with peroxidase-labeled biotinylated anti-mouse TNF-α, IL-6, and KC monoclonal secondary antibodies in assay diluents for 1 h. Finally, the plates were treated with TMB substrate solution (KPL, San Diego, CA, USA) for 30 min, and the reaction was stopped by the addition TMB stop solution. Optical density was measured at 450 nm in a microplate reader (SOFT max PRO, version 3.1 software, Sunnyvale, CA, USA). The detection limits for the TNF-α, IL-6, and KC ELISAs were 1000 pg/ml each. The optical densities for TNF-α, IL-6, and KC were each divided by the total protein concentrations of the respective BALF samples for standardization purposes.

### Histological examination of lung tissues

The lung tissues were removed from the mice, and the right, lower lobes of the lungs were removed for histological analysis. Four percent paraformaldehyde fixing solution was infused into the lungs. The specimens were dehydrated and embedded in paraffin. For histological examination, 4-μm sections of embedded tissues were cut on a rotary microtome, after which they were placed on glass slides, deparaffinized, and stained sequentially with hematoxylin and eosin (H&E). Images of lung tissue sections stained with H&E were acquired with an Olympus BX51 microscope (Olympus, Tokyo, Japan) under × 200 magnification. Five randomly selected sections of the slides, cross-sections of lung parenchyma, were captured, digitized and evaluated using Image Pro-Plus 5.1 software (Media Cybernetics, Inc., Silver Spring, MD, USA). Mean alveolar airspace (MAA), a quantitative assessment of lung structure, was determined from the sum of the alveolar airspace areas divided by the number of identified alveoli. The bronchial epithelium was observed with periodic acid Schiff (PAS)-stained sections.

### Statistical analysis

Statistical analysis of the data was conducted using Prism 5 software (GraphPad Software Inc., Irvine, CA, USA). All values are presented as the means ± S.E.M. (standard error of the mean). Differences between the means of the control and the treatment samples were determined by one-way ANOVA for multiple group comparisons followed by Newman-Keuls post hoc comparisons. In all cases, *p* < 0.05 was considered to be statistically significant.

## Results

### Stemona tuberosa extract (ST) inhibits the increase of inflammatory cells in BALF

Stemona granule was analyzed using HPLC-ESI-MS for the analysis and identification of major components. As shown in Table 1, characteristic components of *Stemona* species including stemona alkaloids were identified by the comparison of their retention order and molecular weight with previous reports [24].

**Table 1:** **The observed and calculated mass numbers ofLC/ESI-MSpeaks of Stemonae Radix granules**

Peak No.	Rt (min)	Theoretical mass [M + H] ^+^	Observed mass [M + H] ^+^	Mass difference (mmu)	Identification
1	3.073	406.22239	406.22642	-4.03	Tuberostemoninol/Tuberostemonone
2	5.658	338.19618	338.19668	-0.50	6-Hydroxycroomine/Tuberospironine
3	15.040	392.24313	392.24367	-0.54	N-Oxytuberostemonine
4	17.402	376.24821	376.24806	0.15	Tuberostemonine N
5	19.311	390.22748	390.23615	-8.67	Stemoninine
6	25.729	376.24821	376.24755	0.66	Tuberostemonine K/Neotuberostemonine/Tuberostemonine/ Tuberostemonine H

### Stemona tuberosa extract (ST) inhibits the increase of inflammatory cells in BALF

**Figure 3:**
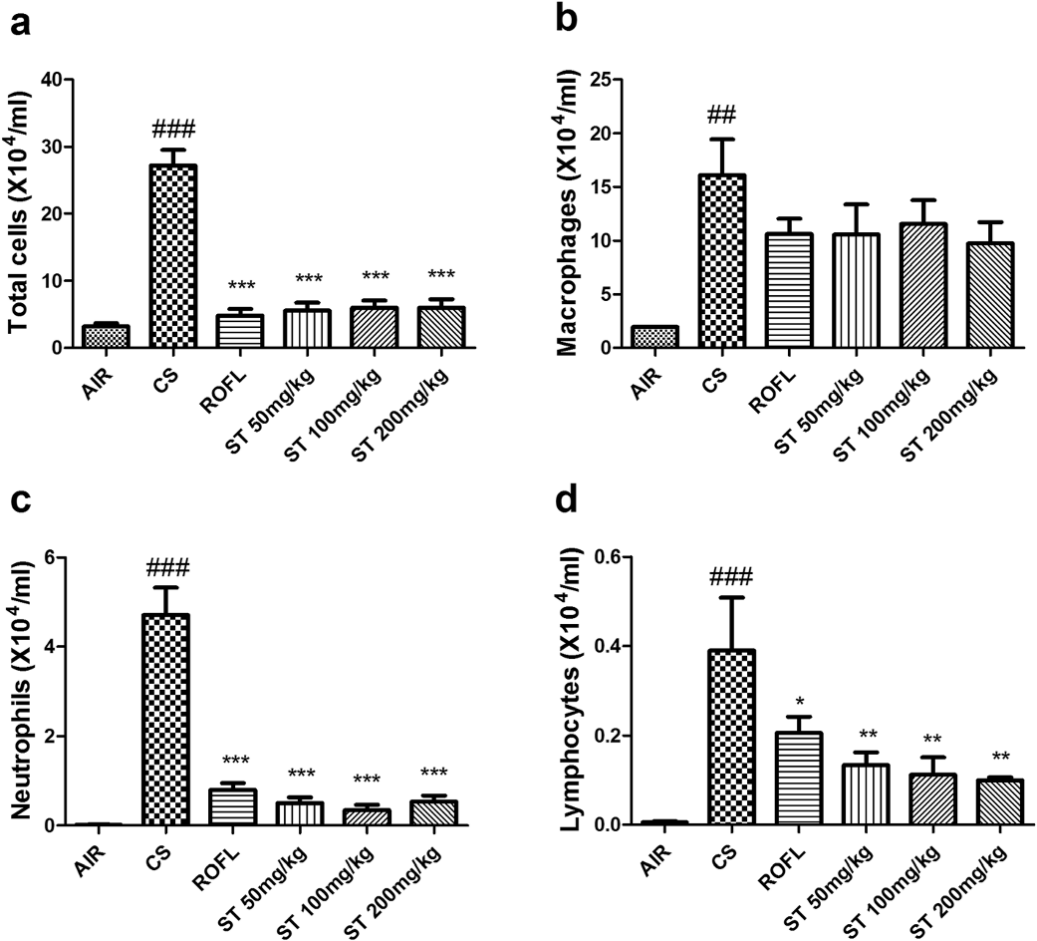
**Total and differential cell counts in BALF.** Exposure to cigarette smoke (CS) increased the numbers of total cells, macrophages, neutrophils, and lymphocytes in BALF. In addition, ST significantly reduced the numbers of these inflammatory cells in BALF to a similar degree as roflumilast. The numbers of cells in BALF were counted using a hemocytometer, and differential cell counts were performed on slides prepared by cytocentrifugation at 250 rpm for 3 min followed by Diff-Quick staining. **a)** Count of total cell number, **b)** Count of macrophages, **c)** Count of neutrophils, **d)** Count of lymphocytes. n = 5 in all groups. Data are shown as the mean ± S.E.M. Statistical analyses were conducted by one-way ANOVA followed by Newman-Keuls Multiple Comparison test; ###*p* < 0.001, ##*p* < 0.01 vs. AIR, ***p < 0.001, **p < 0.01, *p < 0.05 vs. CS.

BALF was analyzed to evaluate how ST has a significant effect on lung inflammation in a subacute CS-induced mouse model. In previous studies, the number of total inflammatory cells was significantly increased when mice were exposed to CS for 2 weeks [23, 25]. In our study, CS caused a significant increase in the number of total inflammatory cells compared with mice that were exposed to fresh air only (Figure 3). On the other hand, roflumilast and ST in all doses showed significant decrease in the number of total inflammatory cells in BALF. It is safe to say that ST has an inhibitory effect on the increase of inflammatory cells in BALF.

An analysis of differential BALF cell counts is also essential to examining the anti-inflammatory effect of ST (Figure 3). Macrophages, neutrophils, and lymphocytes are well known to infiltrate from the alveoli to the bronchoalveolar lavage in order to contribute to lung inflammation that results from CS exposure [26]. The group of mice exposed to CS (CS group) showed significant increases in the number of macrophages, neutrophils, and lymphocytes compared with group of mice that were exposed to fresh air only as a negative control (AIR group). Additionally, roflumilast and ST (50, 100, and 200 mg/kg) inhibited the increase of neutrophils and lymphocytes in BALF compared with the CS group. In detail, the number of macrophages was decreased to a lesser degree than that of neutrophils and lymphocytes. In the previous study, roflumilast did not show the remarkable inhibitory effect on increased macrophages in BALF [13]. ST had the tendency to decrease the number of macrophages in BALF, but this reduction did not surpass that induced by roflumilast. ST also decreased the number of neutrophils to a similar degree as did roflumilast. However, ST surprisingly had a significant effect on decreasing the number of lymphocytes in a dose-dependent manner, and this effect was more significant than that of roflumilast.

### Stemona tuberosa extract (ST) reduces the level of proinflammatory mediators in BALF

**Figure 4:**
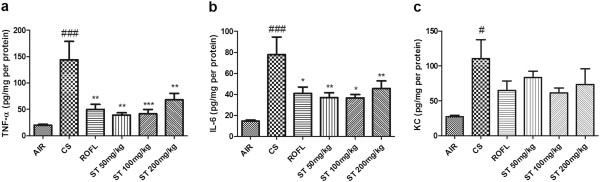
**Measurement of TNF-α, IL-6, and KC levels in BALF by ELISA.** BALF samples were collected as described in Figure 2. ST reduced the level of cytokines (TNF-α, IL-6) and chemokine (KC) when measured with a quantitative sandwich enzyme-linked immunoassay (ELISA). **a)** TNF-α, **b)** IL-6, **c)** KC. n = 5 in all groups. Data are shown as the mean ± S.E.M. Statistical analyses were conducted by one-way ANOVA followed by Newman-Keuls Multiple Comparison test; ###*p* < 0.001, #*p* < 0.05 vs. AIR, ***p < 0.001, **p < 0.01, *p < 0.05 vs. CS.

ELISA was used to evaluate the differences in the expression of inflammatory mediators such as cytokines (TNF-α, IL-6) and the chemokine, KC, between the treatment groups. In previous studies, the secretion of TNF-α and IL-6 into the BALF was shown to be activated in response to CS via some types of inflammatory cells [27–29]. KC, a chemokine which is also called chemokine (C-X-C motif) ligand 1 and growth-related oncogene α, was found to be elevated in lung inflammation induced by CS [30, 31]. In this study, we identified that the levels of both cytokines (TNF-α, IL-6) and KC were significantly elevated in the CS group compared to the AIR group (Figure 4).

Roflumilast, a positive control in this study, had inhibitory effects on the increased cytokine (TNF-α, IL-6) and chemokine (KC) secretion observed in our study (Figure 4). Specifically, the levels of TNF-α and IL-6 in the group of mice treated with roflumilast (ROFL group) were significantly decreased compared with levels in the CS group, but the level of KC only had the tendency to be decreased, as was observed in previous studies [32]. In addition, ST-treated mice showed significantly decreased levels of TNF-α and IL-6 in all treated doses to the same degree as that caused by roflumilast. These remarkable changes in cytokine and chemokine levels strongly demonstrate that ST reduces the level of proinflammatory mediators in BALF.

### Stemona tuberosa extract (ST) disturbs the recruitment of neutrophils in lung tissues

**Figure 5:**
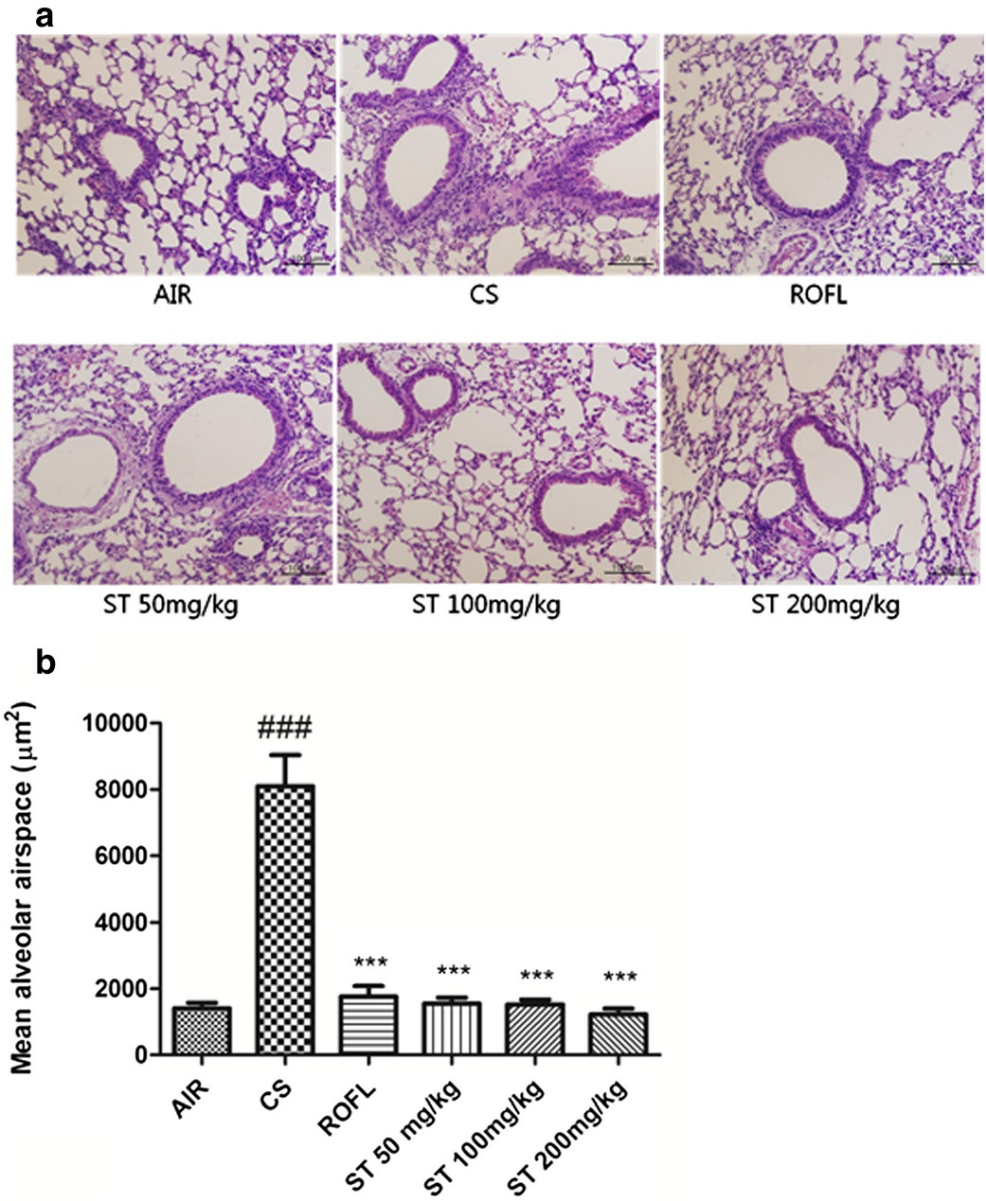
**Neutrophil recruitment and airspace enlargement in lung tissue. a)** The right lower lobes of mice were dissected and stained with hematoxylin and eosin (×200 magnification). ST disturbed the neutrophil accumulation in the peribronchial airway. **b)** Mean alveolar airspace (MAA), a morphometrical analysis, was assessed using Image Pro-Plus 5.1 software as described in the Methods section. n = 5 in all groups. Data are shown as the mean ± S.E.M. Statistical analyses were conducted by one-way ANOVA followed by Newman-Keuls Multiple Comparison test; ###*p* < 0.001 vs. AIR, ***p < 0.001 vs. CS.

We previously found that CS induces an increase of neutrophils in BALF through the assessment of differential cell counts, as described in the Methods section. This recruitment could also be observed upon histological examination. Many researchers have reported that neutrophils accumulate in the tissue to induce the lung inflammation, a leading potential mechanism of alveolar destruction in emphysema [33–35]. The accumulation of neutrophils around the bronchioles of the small airways was shown in lung tissue sections stained with H&E. This accumulation was reported to result from the circulating neutrophils that had marginated into the pulmonary circulation and adhered to endothelial cells in the alveolar wall before infiltrating into the alveolar space [36, 37]. In this study, it was revealed that neutrophils located in the peribronchial airway were predominantly increased in the lung tissues of the CS group compared to the AIR group (Figure 5A). Additionally, the lung morphology of mice treated with ST (50, 100, and 200 mg/kg) showed that ST disturbed the accumulation of neutrophils throughout the peribronchial airway when compared with that of mice that were only exposed to CS without any treatment. The morphological appearance of the ST group was similar to that of the ROFL group.

### Stemona tuberosa extract (ST) decreases the airspace enlargement as observed by morphometric analysis

Several investigations indicated that the exposure to CS significantly induced the destruction of the alveolar wall and airspace enlargement through lung inflammation, oxidative stress and apoptosis [38–42]. We assessed the MAA in lung tissue sections stained with H&E via morphometric analysis, which is a quantitative assessment used to measure the changes of airspace enlargement [43, 44]. Serious airspace enlargement in a subacute CS-induced mouse model due to lung inflammation and alveolar wall destruction could be identified in our study by comparing MAA levels between the AIR group and the CS group (Figure 5B). Meanwhile, the oral administration of roflumilast and all doses of ST significantly reduced the level of MAA. Specifically, the level of MAA in each ST group decreased a little more than that in the ROFL group, and ST had a dose-dependent effect on the prevention of alveolar airspace enlargement, based on this quantitative assessment.

### Stemona tuberosa extract (ST) attenuates the epithelial hyperplasia of the bronchioles

**Figure 6:**
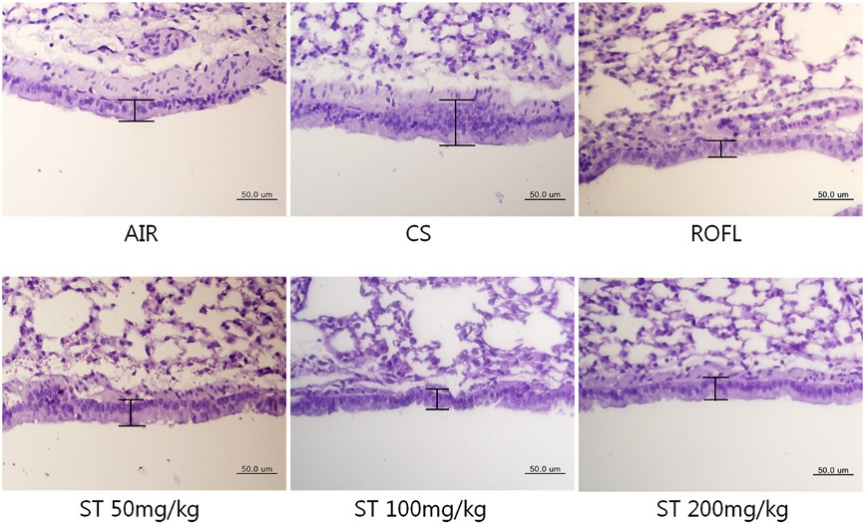
**Epithelial hyperplasia of the bronchioles.** The right lower lobes of mice were dissected and stained with periodic acid Schiff (PAS) as described in the Methods section (×400 magnification). In the cigarette smoke (CS) group, there was a multi-layer of bronchial epithelium, whereas there was approximately a single layer of bronchial epithelium in the fresh air (AIR), roflumilast (ROFL), and ST groups.

Many research articles have described that CS-induced inflammatory cell infiltration, metaplasia of goblet cells, and fibrosis result in the increased thickness of the bronchial epithelium [22, 45–48]. However, even though mice were exposed to CS chronically, the metaplasia of goblet cells in the bronchial epithelium was hardly found, but there were plentiful numbers of goblet cells in human and guinea pig when exposed to CS. Accordingly, some studies of the CS-induced lung inflammation mouse model have only investigated the epithelial hyperplasia of the bronchioles because of the difficulty in estimating the number of goblet cells in the bronchial epithelium of mice [49–51]. PAS stain method has been used to distinguish the epithelial thickness of bronchiole in other studies [22, 52]. In this mouse model of subacute CS-induced lung inflammation, lung tissues of the CS group stained with PAS showed the epithelial hyperplasia of the bronchioles, leading to the increased thickness of bronchial epithelium compared with the AIR group (Figure 6). The inhibition of epithelial hyperplasia of the bronchioles was also observed in PAS-stained lung tissues of the ST group, similar to those of the ROFL group. Morphologically, more than two epithelial cells were clustered and formed a thick layer of bronchial epithelium in that of the CS group, but there was only a single row of epithelial cells in the lung tissues of the ST group.

## Discussion

For thousands of years many drugs including natural plants and chemical compounds have been investigated to find effective treatments for lung diseases such as bronchitis, asthma, and COPD. Particularly in Korean and Chinese medicine, traditional medical doctors have used therapeutic herbs to treat lung diseases according to their traditional medical reference books such as Huangdineijing and DonguiBogam. DonguiBogam was published by the royal physician Jun Heo in the Joseon Dynasty of Korea and designated as UNESCO's memory of the world register in 2009 [53]. Under the contemporary and social requirements, many researchers have made an effort to demonstrate the therapeutic effects of the herbs introduced in those literatures, and found some therapeutic herbs such as *Lilium lancifolium*, *Liriopis tuber*, *Coscinium fenestratum*, and *Prunus armeniaca* have been proved to have an interesting effect on lung diseases [53–56]. ST has also shown promise as an alternative treatment for lung diseases [19, 20].

To evaluate the degree of inhibition of lung inflammation, many researchers prefer the CS-induced mouse model over other models due to the following advantages. This model is induced by the same insult as observed in humans and produces emphysema, airway remodeling, and physiological alterations similar to humans [51]. In our study, the effect of *Stemona tuberosa* on lung inflammation was investigated using a CS-induced mouse model, which is one of the models that is most similar to lung diseases including COPD. Two weeks of cigarette smoke exposure in this model was enough to induce alveolar wall destruction and airspace enlargement, based on previous studies [21, 53–57].

CS, which nearly accounts for 80-90% of COPD cases in the United States, induces pathobiological processes in the respiratory system [58]. A plenty of results from other studies likely demonstrate that airspace enlargement and neutrophil accumulation around the peribronchial airway in our histologic analysis have a significant relationship with the increased macrophages and neutrophils found in BALF after CS exposure [59–63]. However, more detailed studies should be performed to identify whether the inhibitory effect of ST on lung inflammation involves these mechanisms.

In our research, we also investigated the changes in lymphocyte levels as well as those of macrophages and neutrophils because lymphocytes are another important factor in lung inflammation related to respiratory diseases such as asthma and COPD. In asthma, the CD4^+^ T cells, a type of lymphocyte, are the crucial mediators of a Th2 type immune response in the bronchial airway, whereas the CD8^+^ T cells are more important in COPD [64, 65]. The greater the CS exposure, the larger the population of total T cells, including CD3^+^, CD4^+^, CD8^+^, and γδ T cells in the alveolar wall, in the previous study. Of all these T cells, CD8^+^ T cells were the most predominant cell type found in bronchial airways in smokers with emphysema. These results suggested that CD8^+^ T cells induced structural cell apoptosis in the lung [66]. A recent study also demonstrated that CD3^+^ T cells could be another pathogenic cell in mice exposed to CS, leading to neutrophil accumulation, enhanced levels of cytokines and chemokines, structural cell apoptosis of the alveoli, and airspace enlargement [67]. In our research, the increased level of lymphocytes could also be associated with airspace enlargement observed via morphometric analysis. In conclusion, several types of lymphocytes such as CD3^+^, CD4^+^, and CD8^+^ T may be candidate targets of ST, which would explain the finding of decreased lymphocyte levels in BALF and reduced airspace enlargement in the ST groups, compared with those in the CS group.

In our research, we investigated the alteration of the cytokines (TNF-α, IL-6) and the chemokine (KC) in BALF by ELISA to assess the inhibitory effect of ST. These cytokines and the chemokine have been generally used in CS-induced mouse model, especially for COPD in previous studies [21, 68, 69]. Some studies have reported that a variety of cytokines and chemokines in BALF are involved with the lung inflammation induced by CS and are also increased in COPD patients [70]. Previous studies reported that TNF-α overexpression can be observed in the lung tissue of normal mice exposed to CS, whereas TNF-α receptor knockout mice were protected from the severe inflammation caused by CS. Furthermore, increased TNF-α levels stimulated the MMP synthesis of alveolar macrophages, resulting in alveolar wall destruction and airspace enlargement [71, 72]. Meanwhile, IL-6, a cytokine released by macrophages, lymphocytes, and endothelial cells, is another target to treat lung inflammation leading to COPD. IL-6 is also known to contribute to impaired endothelial cell function, which leads to enlarged alveolar airspaces [73, 74]. ST could be a potential alternative to existing treatments that inhibit TNF-α and IL-6 because we have shown in this study that ST can attenuate TNF-α and IL-6 levels.

We investigated the effect of ST on lung inflammation through total cell counts, differential cell counts, changes in the level of the cytokines (TNF-α, IL-6) and the chemokine (KC) by ELISA, and the morphological analysis of lung tissues stained with H&E and PAS. Our goal is to identify a new therapeutic herb for lung diseases such as COPD based on other studies of the respiratory effects of ST [19]. The respiratory effects of ST, however, were demonstrated for symptoms such as antitussive and respiratory depressant effects, so our study tried to minutely examine the immunological changes associated with these effects. In summary, ST inhibited the increase of several inflammatory cells such as macrophages, neutrophils, and lymphocytes, and decreased the levels of proinflammatory cytokines such as TNF-α and IL-6 and the chemokine, KC. This immunological downregulation in lung tissue may lead to reduced alveolar wall destruction, airspace enlargement, and epithelial hyperplasia of the bronchioles, as demonstrated by morphological analysis and MAA measurements. Although inflammatory cells and mediators in BALF and MAA in lung tissue that we investigated were significantly reduced in all doses of ST treatment group rather than CS exposure group, we could not find the differences among the three doses of ST except dose-dependent decrease in the number of lymphocytes. Meanwhile, it is possible that 50 mg/kg of ST, the minimum dose in our study, already reached the maximal effect on lung inflammation in this model.

We could find a few limitations in this mice model to evaluate the effect of ST. First, we had a difficulty in finding the increased number of goblet cells or fibrotic changes in the bronchial epithelium of CS group. To evaluate the effect of ST with these characteristics, researchers should perform this experimental study with other strains of mice, or guinea pig [51, 75]. Secondly, this study was designed to examine the effects of CS exposure in regard to inflammation only. Recent studies, however, examine the effects of CS exposure in the aspect of DNA damage as well as inflammation [76]. Furthermore, one study researched the effect of a therapeutic herb, *Zataria multiflora*, on systemic inflammation of experimental animal model of COPD. They used biochemical parameters such as measurement of malondialdehye and differential cell count in the blood sample [77]. According to these recent studies, other biomarkers that can evaluate the effect of ST on targeting the treatment of COPD should be performed for the further studies.

Previous studies have been conducted to discover the components of ST such as neotuberostemonine, tuberostemonine, croomine, and stemoninine, as well as their chemical structures, effects, and ethnopharmacological uses [20, 24, 78]. In one of these studies, tuberostemonine was proven to have an inhibitory effect on the neuromuscular junction [79]. Additionally, some *Stemona* species other than ST have been continuously used as therapeutic agents in Korean and Chinese medicine. For instance, *Stemona japonica* and its alkaloids have insecticidal and antifungal effects [18, 80]. *Stemona sessilifolia* and its alkaloids also have acetylcholinesterase inhibitory activity as well as antibacterial and antitussive effects [17, 81, 82]. To extend a helping hand to patients with lung diseases such as COPD, the effects of ST should be compared with these similar species through the pathobiological investigation of both experimental and clinical trials. Also, further researches should be performed to discover which phyto-constituents of ST have crucial inhibitory effect on lung inflammation. With the help of additional studies, ST may represent a potential alternative for existing drugs, such as roflumilast, that are used to treat lung diseases and have some side effects. Roflumilast, an oral phosphodiesterase-4 (PDE) inhibitor, can improve lung diseases such as COPD, but side effects have been reported. The most common side effects include diarrhea, nausea, and headache [83]. ST could be a good therapeutic agent for lung diseases and may even be a better treatment than roflumilast. However, it is necessary that the elaborate mechanisms of ST be precisely discovered by additional experiments, and it should be produced in farms of good agricultural practice (GAP) and pharmaceutical companies of good manufacturing practice (GMP) [19]. In conclusion, based on our results that ST has significant inhibitory effects in a mouse model of subacute CS-induced lung inflammation, more studies should be performed to determine the critical targets of ST, whether ST has any side effects, and how ST can be manufactured with the best effectiveness and efficacy for the purpose of its pharmacological development and clinical use.

## Conclusions

We found the significant changes of lung inflammation in a subacute cigarette smoke-induced mouse model. With this mouse model, we discovered that ST treatment decreased a variety of inflammatory cells of the lung such as macrophages, neutrophils, and lymphocytes and reduced the level of cytokines (TNF-α, IL-6) and a chemokine (KC) induced by CS exposure. It also attenuated the airspace enlargement and epithelial hyperplasia of bronchioles in lung tissues obtained from the mouse model of subacute CS-induced lung inflammation. These results strongly suggest that ST may be a new valuable therapeutic agent for the treatment of lung diseases such as COPD.

## References

[1] Sopori M (2002). Effects of cigarette smoke on the immune system. Nat Rev Immunol.

[2] Vestbo J, Sorensen T, Lange P, Brix A, Torre P, Viskum K (1999). Long-term effect of inhaled budesonide in mild and moderate chronic obstructive pulmonary disease: a randomised controlled trial. Lancet.

[3] Nakamura Y, Romberger DJ, Tate L, Ertl RF, Kawamoto M, Adachi Y, Mio T, Sisson JH, Spurzem JR, Rennard SI (1995). Cigarette smoke inhibits lung fibroblast proliferation and chemotaxis. Am J Respir Crit Care Med.

[4] Marwick JA, Kirkham PA, Stevenson CS, Danahay H, Giddings J, Butler K, Donaldson K, Macnee W, Rahman I (2004). Cigarette smoke alters chromatin remodeling and induces proinflammatory genes in rat lungs. Am J Respir Cell Mol Biol.

[5] Randerath E, Miller RH, Mittal D, Avitts TA, Dunsford HA, Randerath K (1989). Covalent DNA damage in tissues of cigarette smokers as determined by 32P-postlabeling assay. J Natl Cancer Inst.

[6] Kier LD, Yamasaki E, Ames BN (1974). Detection of mutagenic activity in cigarette smoke condensates. Proc Natl Acad Sci U S A.

[7] Barnes PJ (2009). The cytokine network in chronic obstructive pulmonary disease. Am J Respir Cell Mol Biol.

[8] Chung KF (2001). Cytokines in chronic obstructive pulmonary disease. Eur Respir J Suppl.

[9] Sin DD, Anthonisen NR, Soriano JB, Agusti AG (2006). Mortality in COPD: Role of comorbidities. Eur Respir J.

[10] Vestbo J, Hurd SS, Agusti AG, Jones PW, Vogelmeier C, Anzueto A, Barnes PJ, Fabbri LM, Martinez FJ, Nishimura M, Stockley RA, Sin DD, Rodriguez-Roisin R (2013). Global Strategy for the Diagnosis, Management, and Prevention of Chronic Obstructive Pulmonary Disease GOLD Executive Summary. Am J Respir Crit Care Med.

[11] Buenestado A, Grassin-Delyle S, Guitard F, Naline E, Faisy C, Israel-Biet D, Sage E, Bellamy JF, Tenor H, Devillier P (2012). Roflumilast inhibits the release of chemokines and TNF-alpha from human lung macrophages stimulated with lipopolysaccharide. Br J Pharmacol.

[12] Field SK (2011). Roflumilast, a Novel Phosphodiesterase 4 Inhibitor, for COPD Patients with a History of Exacerbations. Clin Med Insights Circ Respir Pulm Med.

[13] Martorana PA, Beume R, Lucattelli M, Wollin L, Lungarella G (2005). Roflumilast fully prevents emphysema in mice chronically exposed to cigarette smoke. Am J Respir Crit Care Med.

[14] Rabe KF (2011). Update on roflumilast, a phosphodiesterase 4 inhibitor for the treatment of chronic obstructive pulmonary disease. Br J Pharmacol.

[15] Zheng J, Yang J, Zhou X, Zhao L, Hui F, Wang H, Bai C, Chen P, Li H, Kang J, Brose M, Richard F, Goehring UM, Zhong N (2014). Roflumilast for the treatment of COPD in an Asian population: a randomized, double-blind, parallel-group study. Chest.

[16] Lin LG, Yang XZ, Tang CP, Ke CQ, Zhang JB, Ye Y (2008). Antibacterial stilbenoids from the roots of Stemona tuberosa. Phytochemistry.

[17] Zhang T, Zhang YZ, Tao JS (2007). Antibacterial constituents from Stemona sessilifolia. J Asian Nat Prod Res.

[18] Zhang YZ, Xu GB, Zhang T (2008). Antifungal stilbenoids from Stemona japonica. J Asian Nat Prod Res.

[19] Xu YT, Shaw PC, Jiang RW, Hon PM, Chan YM, But PP (2010). Antitussive and central respiratory depressant effects of Stemona tuberosa. J Ethnopharmacol.

[20] Xu YT, Hon PM, Jiang RW, Cheng L, Li SH, Chan YP, Xu HX, Shaw PC, But PPH (2006). Antitussive effects of Stemona tuberosa with different chemical profiles. J Ethnopharmacol.

[21] Jung KH, Haam KK, Park S, Kim Y, Lee SR, Lee G, Kim M, Hong M, Shin M, Jung S, Bae H (2013). The standardized herbal formula, PM014, ameliorated cigarette smoke-induced lung inflammation in a murine model of chronic obstructive pulmonary disease. BMC Complement Altern Med.

[22] Stebbins KJ, Broadhead AR, Baccei CS, Scott JM, Truong YP, Coate H, Stock NS, Santini AM, Fagan P, Prodanovich P, Bain G, Stearns BA, King CD, Hutchinson JH, Prasit P, Evans JF, Lorrain DS (2010). Pharmacological blockade of the DP2 receptor inhibits cigarette smoke-induced inflammation, mucus cell metaplasia, and epithelial hyperplasia in the mouse lung. J Pharmacol Exp Ther.

[23] Huvenne W, Perez-Novo CA, Derycke L, De Ruyck N, Krysko O, Maes T, Pauwels N, Robays L, Bracke KR, Joos G, Brusselle G, Bachert C (2010). Different regulation of cigarette smoke induced inflammation in upper versus lower airways. Respir Res.

[24] Jiang RW, Hon PM, Zhou Y, Chan YM, Xu YT, Xu HX, Greger H, Shaw PC, But PP (2006). Alkaloids and chemical diversity of Stemona tuberosa. J Nat Prod.

[25] Gualano RC, Hansen MJ, Vlahos R, Jones JE, Park-Jones RA, Deliyannis G, Turner SJ, Duca KA, Anderson GP (2008). Cigarette smoke worsens lung inflammation and impairs resolution of influenza infection in mice. Respir Res.

[26] Wright JL, Hobson J, Wiggs BR, Hogg JC (1988). Comparison of inflammatory cells in bronchoalveolar fluid with those in the lumen and tissue peripheral airways and alveolar airspace. Lung.

[27] Demirjian L, Abboud RT, Li H, Duronio V (2006). Acute effect of cigarette smoke on TNF-alpha release by macrophages mediated through the erk1/2 pathway. Biochim Biophys Acta.

[28] Mortaz E, Kraneveld AD, Smit JJ, Kool M, Lambrecht BN, Kunkel SL, Lukacs NW, Nijkamp FP, Folkerts G (2009). Effect of cigarette smoke extract on dendritic cells and their impact on T-cell proliferation. Plos One.

[29] Soliman DM, Twigg HL (1992). Cigarette smoking decreases bioactive interleukin-6 secretion by alveolar macrophages. Am J Physiol.

[30] Morrison D, Strieter RM, Donnelly SC, Burdick MD, Kunkel SL, MacNee W (1998). Neutrophil chemokines in bronchoalveolar lavage fluid and leukocyte-conditioned medium from nonsmokers and smokers. Eur Respir J.

[31] Traves SL, Culpitt SV, Russell RE, Barnes PJ, Donnelly LE (2002). Increased levels of the chemokines GROalpha and MCP-1 in sputum samples from patients with COPD. Thorax.

[32] Fortin M, D'Anjou H, Higgins ME, Gougeon J, Aube P, Moktefi K, Mouissi S, Seguin S, Seguin R, Renzi PM, Paquet L, Ferrari N (2009). A multi-target antisense approach against PDE4 and PDE7 reduces smoke-induced lung inflammation in mice. Respir Res.

[33] Hunninghake GW, Crystal RG (1983). Cigarette smoking and lung destruction. Accumulation of neutrophils in the lungs of cigarette smokers. Am Rev Respir Dis.

[34] Hoenderdos K, Condliffe A (2013). The neutrophil in chronic obstructive pulmonary disease. Am J Respir Cell Mol Biol.

[35] Noguera A, Batle S, Miralles C, Iglesias J, Busquets X, MacNee W, Agusti AG (2001). Enhanced neutrophil response in chronic obstructive pulmonary disease. Thorax.

[36] Hogg JC, Walker BA (1995). Polymorphonuclear leucocyte traffic in lung inflammation. Thorax.

[37] Pettersen CA, Adler KB (2002). Airways inflammation and COPD: epithelial-neutrophil interactions. Chest.

[38] Farkas L, Farkas D, Warburton D, Gauldie J, Shi W, Stampfli MR, Voelkel NF, Kolb M (2011). Cigarette smoke exposure aggravates air space enlargement and alveolar cell apoptosis in Smad3 knockout mice. Am J Physiol Lung Cell Mol Physiol.

[39] Rahman I, MacNee W (1996). Role of oxidants/antioxidants in smoking-induced lung diseases. Free Radic Biol Med.

[40] Rahman I, Biswas SK, Kode A (2006). Oxidant and antioxidant balance in the airways and airway diseases. Eur J Pharmacol.

[41] Imai K, Mercer BA, Schulman LL, Sonett JR, D'Armiento JM (2005). Correlation of lung surface area to apoptosis and proliferation in human emphysema. Eur Respir J.

[42] Aoshiba K, Yokohori N, Nagai A (2003). Alveolar wall apoptosis causes lung destruction and emphysematous changes. Am J Respir Cell Mol Biol.

[43] Goncharova EA, Goncharov DA, Fehrenbach M, Khavin I, Ducka B, Hino O, Colby TV, Merrilees MJ, Haczku A, Albelda SM, Krymskaya VP (2012). Prevention of alveolar destruction and airspace enlargement in a mouse model of pulmonary lymphangioleiomyomatosis (LAM). Sci Transl Med.

[44] Hsia CC, Hyde DM, Ochs M, Weibel ER (2010). An official research policy statement of the American Thoracic Society/European Respiratory Society: standards for quantitative assessment of lung structure. Am J Respir Crit Care Med.

[45] Saetta M, Di Stefano A, Turato G, Facchini FM, Corbino L, Mapp CE, Maestrelli P, Ciaccia A, Fabbri LM (1998). CD8+ T-lymphocytes in peripheral airways of smokers with chronic obstructive pulmonary disease. Am J Respir Crit Care Med.

[46] Saetta M, Turato G, Baraldo S, Zanin A, Braccioni F, Mapp CE, Maestrelli P, Cavallesco G, Papi A, Fabbri LM (2000). Goblet cell hyperplasia and epithelial inflammation in peripheral airways of smokers with both symptoms of chronic bronchitis and chronic airflow limitation. Am J Respir Crit Care Med.

[47] Vlahovic G, Russell ML, Mercer RR, Crapo JD (1999). Cellular and connective tissue changes in alveolar septal walls in emphysema. Am J Respir Crit Care Med.

[48] Zhu Z, Lee CG, Zheng T, Chupp G, Wang J, Homer RJ, Noble PW, Hamid Q, Elias JA (2001). Airway inflammation and remodeling in asthma. Lessons from interleukin 11 and interleukin 13 transgenic mice. Am J Respir Crit Care Med.

[49] Luppi F, Aarbiou J, van Wetering S, Rahman I, de Boer WI, Rabe KF, Hiemstra PS (2005). Effects of cigarette smoke condensate on proliferation and wound closure of bronchial epithelial cells in vitro: role of glutathione. Respir Res.

[50] Jeffery PK, Ayers M, Rogers D (1982). The mechanisms and control of bronchial mucous cell hyperplasia. Adv Exp Med Biol.

[51] Wright JL, Cosio M, Churg A (2008). Animal models of chronic obstructive pulmonary disease. Am J Physiol Lung Cell Mol Physiol.

[52] Tan YF, Zhang W, Yang L, Jiang SP (2011). The effect of formoterol on airway goblet cell hyperplasia and protein Muc5ac expression in asthmatic mice. Eur Rev Med Pharmacol Sci.

[53] Lee E, Yun N, Jang YP, Kim J (2013). Lilium lancifolium Thunb. extract attenuates pulmonary inflammation and air space enlargement in a cigarette smoke-exposed mouse model. J Ethnopharmacol.

[54] Lee YC, Lee JC, Seo YB, Kook YB (2005). Liriopis tuber inhibit OVA-induced airway inflammation and bronchial hyperresponsiveness in murine model of asthma. J Ethnopharmacol.

[55] Do JS, Hwang JK, Seo HJ, Woo WH, Nam SY (2006). Antiasthmatic activity and selective inhibition of type 2 helper T cell response by aqueous extract of semen armeniacae amarum. Immunopharmacol Immunotoxicol.

[56] Rai RV, Rajesh PS, Kim H (2013). Medicinal use of Coscinium fenestratum (Gaertn.) Colebr.: an short review. Orient Pharm Exp Med.

[57] Valenca SS, Castro P, Pimenta WA, Lanzetti M, Silva SV, Barja-Fidalgo C, Koatz VL, Porto LC (2006). Light cigarette smoke-induced emphysema and NFkappaB activation in mouse lung. Int J Exp Pathol.

[58] Sethi JM, Rochester CL (2000). Smoking and chronic obstructive pulmonary disease. Clin Chest Med.

[59] Tetley TD (1993). New perspectives on basic mechanisms in lung disease. 6. Proteinase imbalance: its role in lung disease. Thorax.

[60] Shapiro SD (1999). The macrophage in chronic obstructive pulmonary disease. Am J Respir Crit Care Med.

[61] D'Hulst AI, Vermaelen KY, Brusselle GG, Joos GF, Pauwels RA (2005). Time course of cigarette smoke-induced pulmonary inflammation in mice. Eur Respir J.

[62] Molet S, Belleguic C, Lena H, Germain N, Bertrand CP, Shapiro SD, Planquois JM, Delaval P, Lagente V (2005). Increase in macrophage elastase (MMP-12) in lungs from patients with chronic obstructive pulmonary disease. Inflamm Res.

[63] Ramos C, Cisneros J, Gonzalez-Avila G, Becerril C, Ruiz V, Montano M (2009). Increase of matrix metalloproteinases in woodsmoke-induced lung emphysema in guinea pigs. Inhal Toxicol.

[64] Barnes PJ (2001). Th2 cytokines and asthma: an introduction. Respir Res.

[65] Freeman CM, Han MK, Martinez FJ, Murray S, Liu LX, Chensue SW, Polak TJ, Sonstein J, Todt JC, Ames TM, Arenberg DA, Meldrum CA, Getty C, McCloskey L, Curtis JL (2010). Cytotoxic potential of lung CD8(+) T cells increases with chronic obstructive pulmonary disease severity and with in vitro stimulation by IL-18 or IL-15. J Immunol.

[66] Majo J, Ghezzo H, Cosio MG (2001). Lymphocyte population and apoptosis in the lungs of smokers and their relation to emphysema. Eur Respir J.

[67] Motz GT, Eppert BL, Wesselkamper SC, Flury JL, Borchers MT (2010). Chronic cigarette smoke exposure generates pathogenic T cells capable of driving COPD-like disease in Rag2-/- mice. Am J Respir Crit Care Med.

[68] Brandsma CA, Hylkema MN, Luinge MA, Geerlings M, Klok PA, Cassee FR, Timens W, Postma DS, Kerstjens HA (2008). Nitrogen dioxide exposure attenuates cigarette smoke-induced cytokine production in mice. Inhal Toxicol.

[69] McGrath-Morrow SA, Lauer T, Collaco JM, Yee M, O'Reilly M, Mitzner W, Neptune E, Wise R, Biswal S (2011). Neonatal hyperoxia contributes additively to cigarette smoke-induced chronic obstructive pulmonary disease changes in adult mice. Am J Respir Cell Mol Biol.

[70] Gessner C, Scheibe R, Wotzel M, Hammerschmidt S, Kuhn H, Engelmann L, Hoheisel G, Gillissen A, Sack U, Wirtz H (2005). Exhaled breath condensate cytokine patterns in chronic obstructive pulmonary disease. Respir Med.

[71] Chung KF (2006). Cytokines as targets in chronic obstructive pulmonary disease. Curr Drug Targets.

[72] Churg A, Wang RD, Tai H, Wang X, Xie C, Wright JL (2004). Tumor necrosis factor-alpha drives 70% of cigarette smoke-induced emphysema in the mouse. Am J Respir Crit Care Med.

[73] Barnes PJ, Celli BR (2009). Systemic manifestations and comorbidities of COPD. Eur Respir J.

[74] Bucchioni E, Kharitonov SA, Allegra L, Barnes PJ (2003). High levels of interleukin-6 in the exhaled breath condensate of patients with COPD. Respir Med.

[75] Bartalesi B, Cavarra E, Fineschi S, Lucattelli M, Lunghi B, Martorana PA, Lungarella G (2005). Different lung responses to cigarette smoke in two strains of mice sensitive to oxidants. Eur Respir J.

[76] Itoh M, Tsuji T, Nakamura H, Yamaguchi K, Fuchikami J, Takahashi M, Morozumi Y, Aoshiba K (2014). Systemic effects of acute cigarette smoke exposure in mice. Inhal Toxicol.

[77] Boskabady MH, Gholami Mhtaj L (2014). Effect of the Zataria multiflora on Systemic Inflammation of Experimental Animals Model of COPD. Biomed Res Int.

[78] Zhou Y, Jiang RW, Hon PM, Xu YT, Chan YM, Chan TW, Xu HX, Ding LS, But PP, Shaw PC (2006). Analyses of Stemona alkaloids in Stemona tuberosa by liquid chromatography/tandem mass spectrometry. Rapid Commun Mass Spectrom.

[79] Shinozaki H, Ishida M (1985). Inhibitory actions of tuberostemonine on the excitatory transmission at the crayfish neuromuscular junction. Brain Res.

[80] Tang CP, Chen T, Velten R, Jeschke P, Ebbinghaus-Kintscher U, Geibel S, Ye Y (2008). Alkaloids from stems and leaves of Stemona japonica and their insecticidal activities. J Nat Prod.

[81] Lai DH, Yang ZD, Xue WW, Sheng J, Shi Y, Yao XJ (2013). Isolation, characterization and acetylcholinesterase inhibitory activity of alkaloids from roots of Stemona sessilifolia. Fitoterapia.

[82] Yang XZ, Zhu JY, Tang CP, Ke CQ, Lin G, Cheng TY, Rudd JA, Ye Y (2009). Alkaloids from roots of Stemona sessilifolia and their antitussive activities. Planta Med.

[83] Calverley PM, Sanchez-Toril F, McIvor A, Teichmann P, Bredenbroeker D, Fabbri LM (2007). Effect of 1-year treatment with roflumilast in severe chronic obstructive pulmonary disease. Am J Respir Crit Care Med.

[CR84] The pre-publication history for this paper can be accessed here:http://www.biomedcentral.com/1472-6882/14/513/prepub

